# Identifying Selection in the Within-Host Evolution of Influenza Using Viral Sequence Data

**DOI:** 10.1371/journal.pcbi.1003755

**Published:** 2014-07-31

**Authors:** Christopher J. R. Illingworth, Andrej Fischer, Ville Mustonen

**Affiliations:** 1 Department of Genetics, University of Cambridge, Cambridge, United Kingdom; 2 Wellcome Trust Sanger Institute, Hinxton, Cambridge, United Kingdom; University of Texas at Austin, United States of America

## Abstract

The within-host evolution of influenza is a vital component of its epidemiology. A question of particular interest is the role that selection plays in shaping the viral population over the course of a single infection. We here describe a method to measure selection acting upon the influenza virus within an individual host, based upon time-resolved genome sequence data from an infection. Analysing sequence data from a transmission study conducted in pigs, describing part of the haemagglutinin gene (HA1) of an influenza virus, we find signatures of non-neutrality in six of a total of sixteen infections. We find evidence for both positive and negative selection acting upon specific alleles, while in three cases, the data suggest the presence of time-dependent selection. In one infection we observe what is potentially a specific immune response against the virus; a non-synonymous mutation in an epitope region of the virus is found to be under initially positive, then strongly negative selection. Crucially, given the lack of homologous recombination in influenza, our method accounts for linkage disequilibrium between nucleotides at different positions in the haemagglutinin gene, allowing for the analysis of populations in which multiple mutations are present at any given time. Our approach offers a new insight into the dynamics of influenza infection, providing a detailed characterisation of the forces that underlie viral evolution.

## Author Summary

The evolution of the influenza virus is of great importance for human health. Through evolution, current influenza viruses develop the ability to infect people who have been vaccinated against earlier strains. New strains of influenza that infect birds and pigs could evolve to infect and spread between people, causing a global pandemic. The influenza virus lives within a human or animal host, so that viral evolution happens within, or in the spread between, individuals. As such, what happens to the virus during the course of an infection is a question of great interest. We here describe a statistical method that uses viral genome sequence data to measure how evolution affects the influenza virus within a single host. Studying data from infections transmitted between pigs, we find evidence for evolutionary adaptation in six of sixteen animals for which data were available. In one case, an immune response mounted by a pig against the virus is apparent. Our method provides a statistical framework for using sequence data to study viral evolution on very short timescales, enabling new research into within-host viral evolution.

## Introduction

The overall risk to human health posed by the novel H7N9 influenza virus [1], while potentially severe, is as yet unknown [2], [3]. Pandemic influenza is a zoonosis [4], and as such any new pandemic may be expected to arise through a two-step process [5], [6], the virus first gaining the ability to cause sporadic, localised infections in humans until, after a second transition, emerging into a global pandemic. Each of these steps are evolutionary in nature, being characterised in turn by the adaptation of a virus to be able to infect a human host, and the development of increased transmissibility between hosts. In the nH7N9 strain, the first of these steps has already taken place, including the acquisition of mutations responsible for human-specific receptor binding [7]. Progression to a global epidemic, therefore, depends upon the evolution of increased transmissibility of the virus, a phenotypic change which can only occur while the virus grows in a host environment. As is true for other viral species [8], understanding the intra-host evolution of influenza is an important task.

A vast array of mathematical modelling approaches have been directed at the questions of influenza infection, transmission, and evolution [9]. Of particular relevance to this study are models which track the dynamics of a single infection. Based upon observed changes in viral titre over time, inferences have been made of many important properties of infection, including the reproductive number for cellular infection, the timescale and numbers of viruses produced during the infection of a cell, and the impact upon the viral population of both innate and adaptive immune responses [10]–[14]. Considering data of intracellular RNA levels, the fine detail of viral replication within a cell has been described [15]. Evolutionary models of competition between viral strains have clarified the relationship between selection for growth and transmission effects, and the dynamics of immune escape [16]–[18].

In the cases above, the viral population was either modelled as a population of identical individuals, or as a set of distinct classes of virus, characterised by differing immune escape or transmission properties. Building upon these approaches, a genetic classification of viruses was used to model H5N1 influenza evolution [19]; the fitness of a virus was defined according to the presence or absence of a set of mutations. Here we divide the viral population in a similar manner, expressing the fitness of a virus as a function of its genetic composition. However, rather than analysing the consequences of a proposed fitness landscape, we here infer how selection was actually at work based upon observed genetic sequence data.

In chronic infections such as HIV, time-resolved sequence data from individual hosts is readily available [20]. However, the course of an influenza infection, even in an immunocompromised host [21], is relatively short. As such, time-resolved genetic data is rare, the main examples having been collected from experimentally-infected animal populations [22], [23]. In this work, we consider data from one such study, examining the evolution of H1N1 influenza within individuals in a swine population [24], [25].

The basic principle of our method is to learn the role of selection acting upon a viral population by means of a maximum likelihood method. We adopt a coarse-grained quasispecies model (cf. [26]) to describe the evolution of the viral population, in which viruses are classified according to the nucleotides (here denoted alleles) present at a limited number of positions (or loci) in their genomic sequence. In this model, evolution proceeds deterministically, contingent only upon the initial state of the population, and the role of selection for or against specific alleles. By considering the consequences for the population dynamics of different proposed models of selection, and comparing these to the observed evolution of the system, we estimate how selection was at work.

The low rate of recombination within RNA segments of influenza [27], [28], combined with a high viral mutation rate, leads to complex evolutionary dynamics, with the fate of mutations being strongly affected by genetic hitchhiking and clonal interference [29]–[31]. As such, discerning the effects of selection requires that interactions between alleles at different loci are taken into account [32]. Here this is achieved by considering the frequencies of haplotypes, sets of sequences with specific alleles at specific loci (e.g. allele C at locus *i* and allele T at locus *j*).

In our model, the viral population can be described at potentially any genomic resolution, keeping track of the population in terms of haplotyes spanning arbitrary numbers of loci. However, higher-locus models are more computationally demanding. As such, we first apply a filtering process to cut out loci at which alleles do not show statistical evidence of having evolved under selection. For each polymorphic locus, we use a single-locus model of evolution to find alleles that appear to evolve in a non-neutral behaviour, changing in frequency over time. Change in the frequency of an allele may occur as the direct result of selection, or due to linkage disequilibrium with a selected allele, or alleles, at other loci. As such, to distinguish between these cases, wherever apparent non-neutrality is observed at more than one locus, we apply a multi-locus model of haplotype frequency change to the data. This model explicitly accounts for interactions between alleles at different loci, and is used to identify the maximum likelihood explanation for the changes observed in the sequence data.

As has been noted elsewhere, the use of viral sequence data to understand population structures requires substantial care (e.g. [33], [34]). Selective amplification of sequences, or general sequencing bias, can produce a misleading picture of a population as a whole. PCR-induced recombination can lead to false measurements of linkage disequilibrium between alleles at different loci. We discuss the potential impact of each of these factors upon our results.

## Results

**Figure 1:**
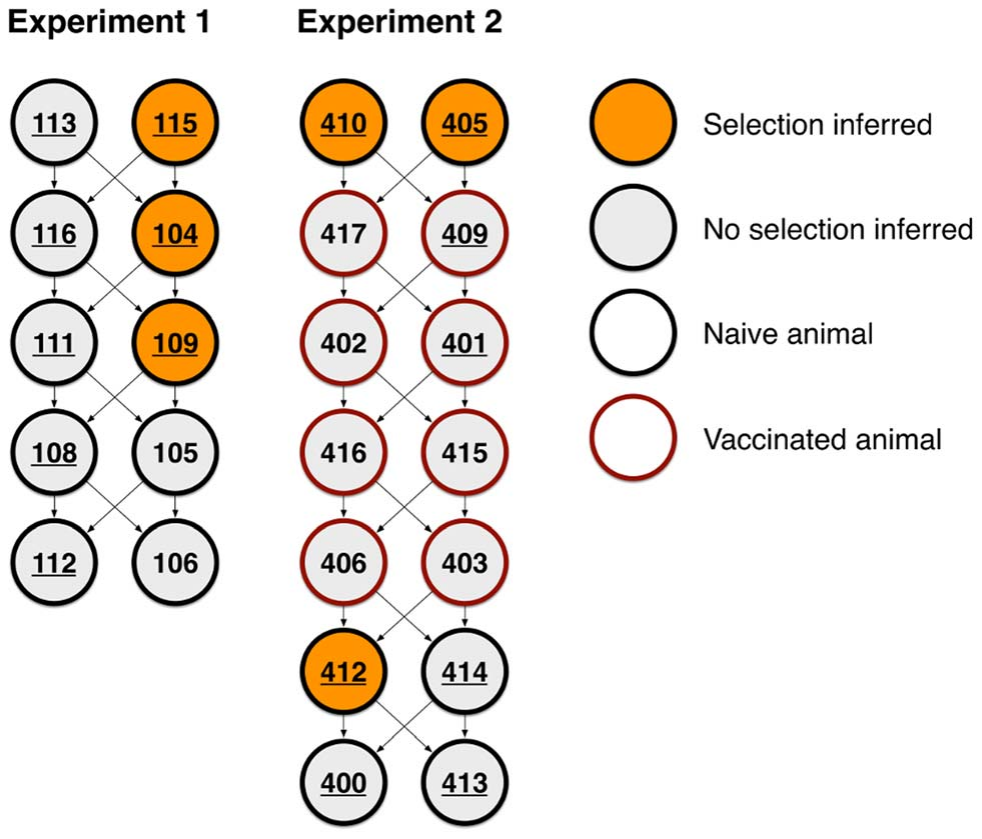
Evidence for selection was found in viruses from six animals across two experiments. Each pig is represented by a circle, numbered by index. Arrows between pigs represent potential transmission events. Pigs vaccinated before being infected are outlined in red; non-vaccinated pigs are outlined in black. Numbers of pigs for which data about the viral population was available for more than one time-point are underlined. Pigs in which selection was identified are highlighted in orange.

Viral sequence data collected from a previous transmission experiment [25] were analysed. An overview of the structure of this experiment is shown in Figure 1. The chain of infection was propagated by a process of housing pairs of uninfected pigs with pairs of infected pigs, the previously-infected pigs being removed after transmission had occurred. Throughout the experiment, samples were collected from pigs using nasal swabs, with viral sequences being amplified via RT-PCR and Sanger sequenced. Viral sequences were collected from the majority of the pigs; for 16 of the 24 pigs involved in the experiment, data was collected at more than one time-point, an essential prerequisite for our method. For the samples collected in these animals the depth of sequencing varied from 6 to 81 sequences (mean 51) from a pig at a given time-point, with data being collected at up to five time-points across the course of an infection. Limited transmission of variants was observed between individual infections.

**Figure 2:**
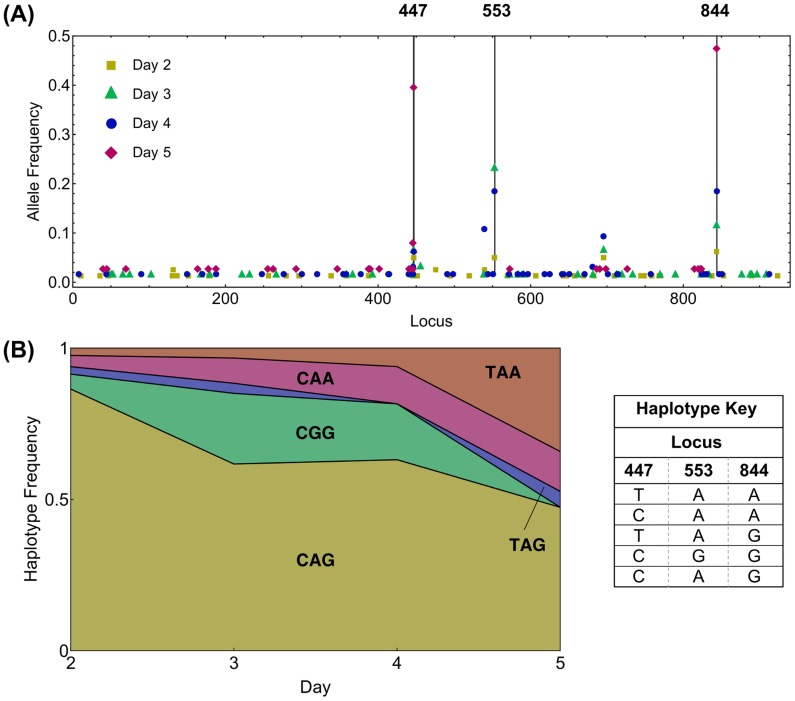
Observations of the viral population in Pig405. **(A)** Minor allele frequencies greater than zero recorded at each locus over time. Vertical black lines indicate loci at which apparent non-neutral behaviour in a minor allele frequency was found; indices for these loci are displayed above the figure. At locus 844 a continual increase in the minor allele frequency over time can be seen; at locus 553 the minor allele frequency increases between days 2 and 3, then decreases to zero. At locus 447, the frequency of the minority allele reaches a value close to 0.4 at the final time-point; a lower allele frequency is seen at the adjacent locus 446. **(B)** Haplotype frequencies for alleles at the three loci that exhibit non-neutral behaviour.

**Figure 3:**
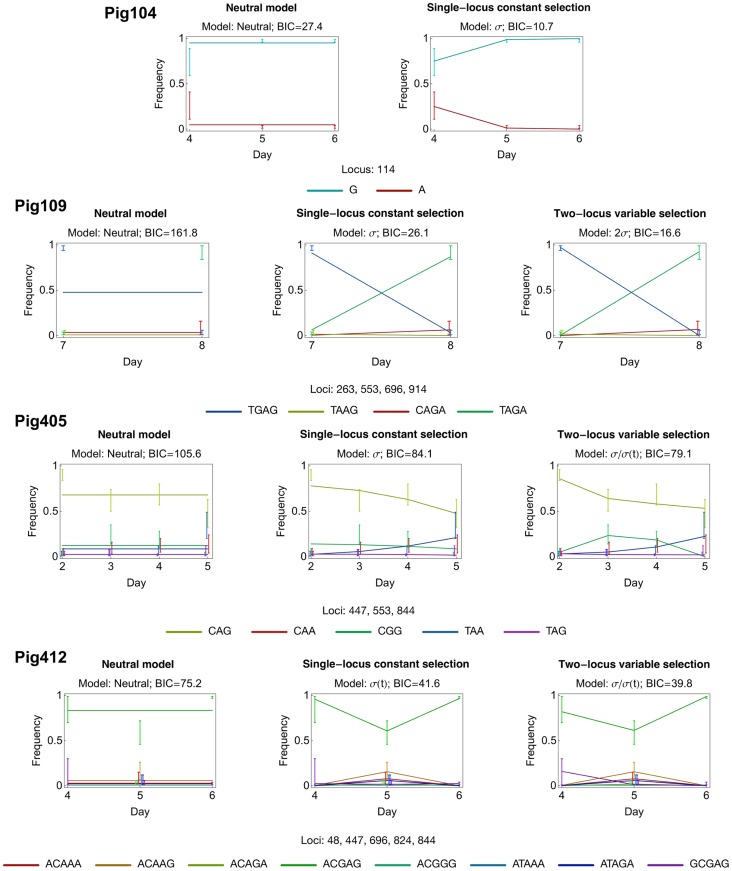
Representative haplotype frequency plots for different models of selection. Comparative model fits for **Pig104:** A model of constant selection against the G→A mutation at locus 113 outperforms the neutral model, **Pig109:** A model in which two of three mutations at loci 553, 696, and 914 outperforms models in which one or none of these mutations is under selection. **Pig405:** A two-locus selection model of constant selection for the G→A mutation at locus 844, with variable, decreasing selection for the A→G mutation at locus 553 gave the best fit to the data. **Pig412:** A model of time-dependent selection at the locus 696, with constant negative selection at locus 48 was optimal. Coloured error bars show 95% confidence intervals for the marginal frequency of each haplotype given the observation; error bars are offset from their respective time-points to allow their identification. Inferences are shown only for haplotypes that were observed in the sequence data. In Pig405 the CAA haplotype frequency is obscured below the TAA frequency.

In our analysis, non-neutral behaviour was identified in six populations. In general, signs of selection were relatively rare. While very many individual mutations were observed in the population as a whole, most of the substantial changes in allele frequency occurred at a small number of sites (e.g. Figure 2). As such, eighteen alleles in the dataset were identified as being potentially non-neutral. Interference effects between alleles were found to be of importance; of these eighteen alleles, a total of nine were identified as being genuinely under selection, changes in frequency at the other nine being explicable in terms of linkage disequilibrium with other selected alleles. In the populations identified to be non-neutral, a variety of forms of selection were found, including evidence for time-dependent selection, and for selection acting simultaneously at more than one locus (a selection of inferred trajectories are shown in Figure 3; further inferences are presented in Supporting Figure S1). Our multi-locus model discriminated between cases where multiple alleles changed in frequency under independent selection, and cases where selection acting upon one allele led to substantial changes in the frequency of others (Table 1).

**Table 1:** Likelihood data for selected models.

Pig	Model	Potential driver(s)							Log L	BIC
		Selection coefficients								
104		**114**								
104	Neutral	0							−13.7	37.6
104	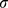	−1.6							−2.8	**21.0**
104	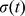	(−1.6, −12.5^*^)							−2.8	26.0
109		**263**	**553**	**696**		**914**		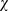		
109	Neutral	0	0	0		0		0	−80.9	180.7
109	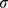	0	0	0		3.0		0	−10.7	44.9
109	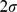	0	0	2.8		2.8		0	−3.6	**35.5**
113		**447**			**824**		**844**	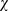		
113	Neutral	0			0		0	0	−3.8	**7.7**
113	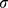	0			0		1.0	0	−1.8	8.2
115		**188**								
115	Neutral	0							−4.9	19.9
115	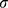	1.2							−1.7	**18.3**
115	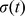	(−0.3, 2.8)							−1.5	23.0
405		**447**			**553**		**844**	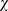		
405	Neutral	0			0		0	0	−52.8	133.1
405	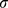	0			0		0.4	0	−39.3	111.6
405	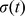	0			0		(0.3, 0.3, 0.7)	0	−38.5	121.0
405	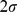	0.3			0		0.3	0	−37.3	113.1
405	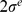	0.1			0		0.3	0.2	−36.9	117.8
405	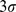	0.3			0.1		0.3	0	−36.6	117.3
405	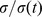	0			(0.9, −0.1, −6.7^*^)		0.4	0	−28.6	**106.6**
405	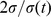	0.2			(0.9, −0.1, −4.7^*^)		0.3	0	−26.7	108.4
405	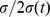	(0.3, 0.0, −0.2)			(0.9, −0.1, −2.5^*^)		0.3	0	−24.2	114.4
410		**447**								
410	Neutral	0							−10.7	31.9
410	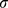	0.0							−10.6	37.2
410	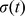	(−1.2, 1.3)							−4.9	**31.1**
412		**48**	**447**	**696**		**824**	**844**	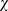		
412	Neutral	0	0	0		0	0	0	−37.6	114.7
412	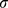	0	0	−0.5		0	0	0	−33.7	111.8
412	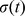	0	0	(2.2, −11.0^*^)		0	0	0	−15.9	81.1
412	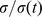	−1.2	0	(1.8, −22.8^*^)		0	0	0	−12.5	**79.3**

The optimal model of each type is given in each case. Model codes are 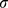: Constant selection at a single locus; 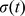: Time-dependent selection at a single locus; 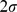: Additive selection at two loci; 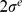: Epistatic selection at two loci (where the fitnesses of the 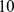 and 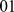 haplotypes at loci 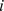 and 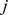 are 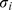 and 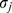 respectively, the fitness of the 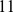 haplotype is 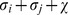); 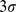: Additive selection at three loci; 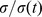: Additive selection at two loci, second locus time-dependent; 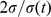: Additive selection at three loci, one time-dependent; 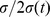: Additive selection at three loci, two time-dependent. Selection coefficients are given in units of 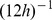. Starred selection coefficients are approximate and could not be determined to high accuracy. The BIC values for the optimal model in each case is displayed in bold. BIC scores are rounded to one decimal place.

In Pig104, strong evidence [35] was found for negative selection acting against the G → A mutation in locus 114, with an inferred selection coefficient of −1.6 per 12 hours (h). Such a magnitude of selection is relatively large; by comparison, an allele at frequency 50% with a selection coefficient of −1 per 12 h would decrease to 12% frequency after one day and to less than 2% after 2 days. The mutation under selection in this case is synonymous, such that the observation of strongly deleterious selection is perhaps a surprising one. While, using our method, no statistical evidence for selection upon this allele was identified in other pigs, the same polymorphism was found in data collected at the earliest time point for pigs 115 and 116, but not at subsequent time-points, consistent with a hypothesis of negative selection for this nucleotide across all viral populations.

In Pig109 strong evidence was found for positive selection upon at least two of three alleles; in favour of the G → A polymorphism at locus 553, the A → G polymorphism at locus 696, or the G → A polymorphism at locus 914. Fixation of all three of these mutations occurred between two samples, and models with any single one of these mutations as the selected allele performed similarly well, giving estimated selection coefficients between 3.0 and 3.1 per 12 h for the selected allele. Joint consideration of four-locus haplotype frequencies provided evidence that at least two of these mutations were independently under selection. The most likely model had coefficients of 2.8 per 12 h at each of the loci 696 and 914. However, the difference between two-locus additive models was small, and models in which any two of the three polymorphisms were under selection performed similarly well (Supporting Table S1). An interesting feature of this result is that the pairs of mutant alleles inferred to be under selection are highly linked, the mutant alleles at loci 696 and 914 appearing only jointly on a sequence, and never in isolation. The inference that selection is acting at two loci, rather than at only one locus, arises from the effect of mutation in the model; this result is explored more fully in Supporting Information. We note that, while the polymorphism at locus 696 is synonymous, those at 553 and 914 are non-synonymous in character, corresponding to the mutations D185N and S305N (the former being contained within the Ca2 epitope region [36]).

In Pig115 weak evidence was found for positive selection in favour of the G → A polymorphism at the locus 188, with an inferred selection coefficient of 1.2 per 12 h. This polymorphism is non-synonymous, representing the amino acid substitution G63E. Bootstrapping of this result against inferences from sequence data that had been randomised in time largely supported this inference; from a total of 200 sets of randomised sequence data, a stronger signal in favour of a model of constant selection was identified in only eight cases. Details of the bootstrapping of all results are given in Supporting Text S1 and in Supporting Figure S2.

In Pig405, strong evidence was found for positive selection acting upon the G →A polymorphism at locus 844, with a selection coefficient of 0.4 per 12 h, along with simultaneous, time-dependent selection acting upon the A → G polymorphism at locus 553. Selection at this second locus was inferred to be initially positive, with mean strength 0.9 per 12 h during the first time-interval, weakly negative during the second time interval, with mean strength −0.1 per 12 h, then finally strongly negative, of mean magnitude greater than −2 per 12 h for the final time interval. Each of these polymorphisms are non-synonymous (corresponding to the mutations V282I and N185D respectively; the mutation at locus 553 is identical to that observed in Pig109, albeit in the reverse direction). Identification of time-dependent selection acting upon the latter, epitope mutation is of particular interest, raising the possibility that this corresponds to an adaptive immune response by the host to the virus. In this population the magnitude of the time-dependent selection inferred for the final time-point was large and negative, but hard to identify with precision. This arises from a time-dependent model of selection being coupled with an observed allele frequency of zero at the final time-point. Excluding the influence of allele frequencies at other loci, the data in such a case can lead to an inference of arbitrarily strong negative selection; the time resolution at which data are collected imposes a limit on the magnitude of selection that can correctly be inferred [37].

In Pig410 we identified weak evidence for time-dependent selection acting upon the synonymous C → T mutation at locus 447; in this case, a bootstrapping calculation produced a stronger signal of selection than that for the real data in only three out of 200 cases (Supporting Figure S2). Time-dependent selection was also identified in Pig412, where strong evidence was found for time-dependent selection acting upon the synonymous G → A mutation at locus 696, with further weak evidence for negative selection acting upon the synonymous A → G mutation at locus 48. Under the multi-locus model, a selection coefficient of 1.8 was identified at locus 696 for the first time interval. The inferred strength of selection at this locus for the second, final time interval was imprecise, but very large and negative; the value of −22.8 per 12 h reported in Table 1 again being caused by an observed frequency of zero at the final time-point.

Alleles at which selection was inferred were distributed across the HA protein (Supporting Figure S3). Significant changes in allele frequency were identified in more than one infection at five different loci (447, 553, 696, 824 and 844). Of these, selection was inferred to act at the loci 696 and 844 in more than one infection. This repetition of mutations may be explained by the design of the experiment; selection is most likely to be observed when polymorphisms exist at non-negligible frequency in the population, while polymorphisms at higher frequencies are more likely to be transmitted between infections.

Under an initial scan for potentially non-neutral alleles, very weak evidence for selection was identified in the data from Pig113 at the three loci 447, 824 and 844. However, under the full multi-locus model, a neutral model of evolution was finally preferred. As we discuss further in Supporting Text S1, our evolutionary model is more conservative in identifying selection in cases where multiple loci are considered simultaneously.

## Discussion

We have here described a novel approach to understanding the within-host evolution of the influenza virus, based upon sequences collected at subsequent times within a single infection. Our method combines a quasispecies model of viral evolution with a hierarchical set of potential models of selection, identifying the evolutionary scenario which best explains the observed sequence data. A crucial component of our model is its accounting for linkage disequilibrium between alleles at different loci; while a single-locus model is sufficient for cases in which only one mutation in a gene changes in frequency [38], the observation of more than one simultaneous change in allele frequency within a non-recombinant gene demands a more sophisticated analysis.

Our approach to inferring selection differs substantially from the calculation of dN/dS [25], not least in considering data at the haplotype frequency level. While in earlier work dN/dS has been applied to sequences collected across viral populations from all observed infections, we allow for the landscape of selection acting upon the virus to vary between animals, or potentially to change within a single animal over time. The results of our analysis also differ; while significant dN/dS ratios were identified at the codon positions 204 and 257, we did not find evidence of selection for alleles at either of these loci. We note that, over short time-scales, difficulties may arise in using numbers of synonymous and non-synonymous mutations to infer selection. While this approach is of great value when applied to diverged sequences, such as those collected from homologous genes in different species [39], its application to sequences from a single population gives results that may be harder to interpret [40], [41].

Our approach to within-host viral evolution is rooted in the interpretation of viral sequence data, collected at multiple times from single infections. By modelling evolution, it is possible to assess the consequences for a viral population of hypothetical fitness landscapes (e.g. [16]). If it is known that a mutation fixes with given probability in a given timescale, the requisite fitness advantage conferred by that mutation can be learnt [42], [43]. However, obtaining a detailed picture of within-host viral evolution requires the use of time-resolved sequencing, describing the population at multiple time points. Our method provides a systematic approach to inferring selection; while the set of potential fitness models is very large [44], we build upwards from a neutral model to increasing complexity, as guided by the data. Keeping data central to our approach means that we may miss the influence of certain fitness effects; sufficient data may not be available to infer the complete picture of how evolution is at work. However, our hierarchical approach means that, given accurate data describing a population, we should not generate false inferences of the presence of selection.

Analysing the data, we identified selection acting upon both synonymous and non-synonymous mutations. Weak selection acting upon synonymous mutations has been identified for codon usage in influenza [45] and against mutations that disrupt RNA structure in HIV [46], although the magnitude of selection inferred here is significantly higher than in either case. While inferring the presence of selection, our method cannot match occurrences of selection to specific biological mechanisms; further data would generally be required to do this.

One result for which a biological mechanism may be proposed is in the viral population of Pig405, where we identified variable, and decreasing selection acting upon a non-synonymous mutation in the Ca2 epitope region, potentially as a result of a specific immune response. For this mutation the timing of the onset of strong negative selection, in the fourth day after exposure to the virus, is earlier than the five days before detection of an adaptive response reported for an H3N2 influenza infection in mice [47]. Further to this, modelling studies have associated the innate immune response with an initial decline in viral load, the adaptive response leading to final clearance of the virus [13]. Here, no drop in viral titre was seen at the time of inferred negative selection, with clearance occurring eight days after infection [24]. Again, further data would be required to produce a more specific conclusion; combined data of viral sequence and immune response would lead to greater understanding of systems such as this.

### Modelling assumptions

Our evolutionary model assumes that the viral population is genetically well-mixed in the host, and that it evolves in a deterministic manner, both with respect to mutation, and to selection. The first of these assumptions asserts that each sample of viruses collected from the pig is representative of the viral population in the animal at the time. This would not be true if, for example, the viral population was split into diverse subsets, with selection acting in very different ways in each. Study of these effects was not possible given the data studied here.

Our assumption of deterministic evolution is based on the underlying viral population being large in number, that is, large enough that 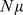 and 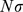 are significantly greater than 1, where *N* is the number of viruses in an animal, *μ* is the mutation rate per locus, and *σ* is the magnitude of selection [48]. Considering selection, the lowest resolution at which we report selection, of 0.1 per 12 h, is, accounting for two rounds of replication in the lifetime of an infected cell [10], [15], equivalent to a fitness difference of 0.05 per generation. As such, this part of the assumption holds if *N* is substantially larger than 20 viruses. Considering mutation, the criterion that 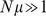 is stricter than that for selection (where *μ* is of order 10^−5^ [49], [50]), requiring *N* to be substantially larger than 10^5^. In influenza, models of replication in a single cell suggest that of the order of 10^4^ virions are produced within each cell [51], while in the samples from which viruses were sequenced, a viral load of between 30 and 5500 particles per *µ*l [24] was measured; once an infection has progressed to the point where viral sequencing is possible, the population is very likely large enough for this to be fulfilled. In the earliest stages of an infection, stochastic mutational behaviour could potentially lead to an incorrect inference of the initial variant frequencies within the population; however, these values are not used to draw any biological conclusions about the system.

Horizontal transmission between co-housed animals was not incorporated into the model; we believe this was unlikely to have greatly influenced the collected data. If the viral populations in the two simultaneously infected pigs were substantially different in composition, transmission of viruses from one animal to the other might alter the composition of the viral population in the second animal. However, the viral populations in this experiment were not sufficiently different in sequence to be able to distinguish superinfection from the growth of *de novo* mutations. Further, while the viral titre implicated in transmission is unknown, we believe that the incoming titre is likely to be substantially smaller than the pre-existing number of viruses in the second infected animal.

### Accuracy of the data

A second assumption in our study is that the collected sequence data are relatively accurate. That is, we assert that the sequences obtained from the sample are representative of the sample itself. The basis of our inference upon data means that the accuracy of the data is vital for obtaining useful results. For example, in addition to raw allele counts, our approach makes explicit use of linkages between mutations. Our method allows for the possibility of generic error in the sequencing process, and fully accounts for the statistical noise inherent to a finite data sample. However, there are systematic data biases that may also affect the results obtained. For example, PCR-induced recombination has the potential to alter the observed frequencies of multi-locus haplotypes [52], [53]. Testing for such an effect, by fitting an exponential model to the observed absolute linkage disequilibrium between pairs of alleles, we found no evidence for such recombination, no decay in this statistic being observed with increasing distance between alleles (Supporting Figure S4).

Sequencing bias also has an effect on whether or not a mutation is recognised as being under selection. Mutations that are preferentially identified by a sequencing method would appear in the sample at higher frequencies, such that changes in their frequencies were amplified, leading to a greater chance that such mutations were found to be under selection. For this dataset, a consistent sequencing method was used to process all of the samples; we therefore assumed sequencing bias to be consistent between samples, such that observed changes in allele frequency were caused either by the finite sampling process, or by a process of mutation and selection. Estimating the extent of sequencing bias in the observed sequences is difficult, the sequences themselves representing the only information about the real viral population. Counting the mutations observed in the data showed a high transition:transversion ratio of 9.7 (Supporting Figure S5). This is broadly consistent with values observed for other RNA viral populations [54], [55], albeit that measurements of this ratio in influenza have previously been based upon global, rather than within-host, populations [56]. Biased sampling, whether occurring via the collection of a biological sample that is unrepresentative of the whole population, or as a result of the subsequent PCR amplification, also has the potential to affect our inference. We have here assumed that the data is an unbiased sample of the real population.

Our inferences are partially limited by the use of sequences describing only the HA1 region of the influenza virus. While our inferences of deviation from neutrality in a population are not affected by alleles elsewhere in the virus, the attribution of selection to given alleles may be affected by unobserved polymorphisms in the HA2 region of influenza, or if reassortment were limited (though see [57]), with alleles in other viral segments. The potential influence of selection acting upon polymorphisms that have not been observed is of greatest relevance to the cases of apparently time-dependent selection; constant selection acting upon interfering mutations causes time-dependent selection effects [32]. One example is the case of Pig412 where initially positive, then negative selection is inferred. In this infection, many haplotypes which are observed at the intermediate time point are no longer seen in the final time point; this pattern is consistent either with a switch in the direction of selection acting upon the synonymous mutation at locus 696, as was inferred, or with very strong positive selection acting upon an unobserved mutation on the consensus haplotype causing a selective sweep later in the observation. Such a scenario is much less likely in the case of Pig405, where the haplotype containing the allele inferred to be under negative selection is outcompeted in the final time interval by four other haplotypes, including that of the initial consensus.

### Conclusions

We have here described a framework for the inference of selection acting upon a viral population within an individual host, based upon time-resolved sequence data. Within-host selection is of importance for the future evolution of the H7N9 influenza virus, and for understanding the epidemiology of other influenza strains. During an epidemic, both within-host growth, and the transmission of viruses, are important, and potentially competing factors; a mutation which is beneficial for within-host growth may prove deleterious for transmission and vice versa. While we have here considered only the first of these factors, our method could easily be used to infer the role of selection for transmission, given specific conditions. First of all, substantial continuity would be required between the native and the transmitted populations, such that changes in allele frequencies before and after transmission were primarily the result of selection; severe bottlenecking would distort the population structure. Secondly, clarity would be required about the source of each infection; in the experiments considered, where an infection begins with an unknown mixture of viruses from two other individuals, the role of selection in transmission cannot be evaluated. Transmission events in the data analysed here have been discussed elsewhere [58]. In more straightforward cases, where transmission occurs between known individuals, and where continuity between viral populations is more evident (e.g. [59]), use of our method to infer selection acting across transmission events is likely to be achievable.

The collection of sequence data describing the within-host evolution of influenza is at present, relatively rare, although we anticipate that improvements in sequencing technology will make such data increasingly accessible. Increased collection of sequence data from patients, and from evolutionary experiments, will greatly add to our understanding of viral infection. Our approach increases the value of such work, characterising in detail the forces that underlie within-host viral evolution.

## Methods

### Description of viral dynamics

Quasispecies theory [26] provides a deterministic description of the evolution of mutation-prone, self-replicating organisms; this framework has profoundly influenced studies of RNA viral evolution [60]–[63]. To describe the evolutionary dynamics of the influenza virus within an individual host we apply a coarse-grained quasispecies model, in which the viral population is described as haplotypes spanning a limited set of loci, rather than as complete viral sequences. Specifically, we represent the viral population as a frequency vector 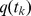, defined at discrete times 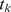, and comprised of elements 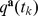, where 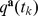 is the fraction of sequences in the population with the haplotype 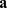; that is, with the nucleotides 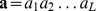 at a subset of loci 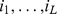 in the viral genome.

To model mutation between haplotypes, we assumed a constant rate of mutation, *μ*, between any two specific nucleotides at a given locus, the probability of mutation from haplotype 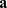 to haplotype 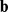 in a single generation being given by 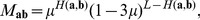
(1)where 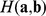 is the Hamming distance between the two haplotype sequences.

Selection was accounted for by ascribing to each haplotype 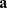 the (potentially time-dependent) selection coefficient 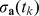. The effect of selection on the haplotype frequency 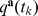 between times 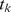 and 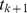 was thus defined by the function 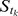: 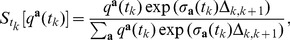
(2)where 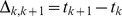. Considering the evolution of influenza, we supposed time-points to be spaced at 12-hour intervals, roughly approximating the time required for a round of intracellular growth within a cell [10]. Within such a round of growth, each virus undergoes two rounds of replication, modelled as having equal mutation rates, with the parameter 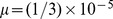 representing an overall rate of mutation per nucleotide per generation of 10^−5^ [49], [50]. Selection was assumed to act upon the viral population once it has exited the cell, giving the relation 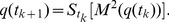
(3)where 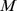 is the matrix consisting of elements 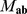, modelling a single round of replication. The behaviour of the system is thus specified in a deterministic manner by the selection parameters 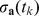, and by the initial state of the system, given by the elements of the vector 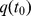.

We note that, while sequence data was collected at known times throughout the course of each infection, the precise moment at which each infection began is unknown. Here, we assumed 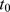 to be precisely 24 hours before the first observed set of sequence data from the infection. While the uncertainty in this value has consequences for the accuracy of the elements of the inferred vector 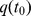, no conclusions were finally drawn from these values.

### Inferring non-neutral behaviour and selection

An inference of selection was carried out by comparing maximum likelihood values obtained under a hierarchical series of models, each specifying the parameters 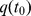 and 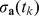. The coarse-grained quasispecies model can be expressed in terms of haplotypes of arbitrary length. We describe the general model below.

#### Evolutionary model

We consider a population with an arbitrary number of polymorphic loci, 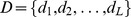, each containing alleles that may or may not be under selection. Each locus may contain one of four nucleotides, such that the vector 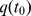, describing the initial state of the population, has 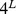 elements, potentially a large number. To reduce the complexity of the model, we therefore considered only the consensus and largest minority alleles at each locus. At each locus, the consensus allele, denoted 0, was defined as the majority allele in the sample collected from the population at the first time of observation. The largest minority allele, denoted 1, was then defined so as to maximise the total number of observed sequences which had one of the 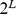 haplotypes 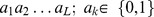 at the loci 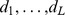. Where more than two alleles were present at a given locus, this simplification distorts the resulting model likelihood. However, in the dataset considered here, such cases were extremely rare; we discuss this further in Supporting Text S1.

Parameters describing 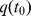 and 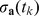 were defined independently. The initial frequency 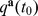 of a haplotype 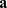 was included as a variable in the model if that haplotype was observed at least once in the sequence data, other initial frequencies being set to zero. Selection parameters were included according to the model of selection being applied. In the basic, neutral model, we set the magnitude of selection 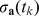 for each haplotype 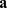 and time point 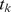 to be zero. In other models of selection, the variant allele could be either neutral, or under constant or time-dependent selection at each locus. Where more than one locus had an allele under selection, the interaction between these alleles could be additive or epistatic in nature. As such, arbitrary models of selection could be considered.

In the model, selection was applied across all relevant haplotypes. In a case of constant selection at a single locus, in which the 1 allele at locus 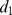 was under selection with magnitude 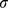, the values 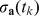 would equal 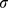 for all haplotypes 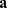 in which the locus 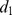 had the allele 1, and for all times 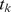.

A final error parameter, 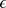, was included, defining the probability that sequencing returns an erroneous haplotype for a given sequence. Assuming that no more than one error occurs in reading any given haplotype (i.e. the error rate is low), we define the haplotype frequency 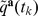, describing the frequency of the haplotype 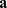 within the model at time 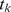, as 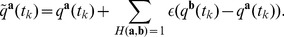
(4)where 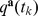 is calculated as described above.

Given a set of parameters describing the initial state of the population, and the selection coefficients acting upon the population, we can write the likelihood of these parameters as 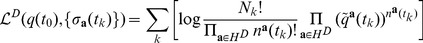
(5)where 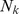 is the total number of samples collected at point 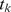, 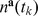 is the number of samples with the haplotype 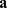 at time 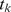, 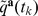 is the predicted frequency of the haplotype 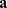 at time 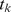, and 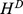 is the set of all haplotypes over the loci in 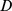.

The Bayesian information criterion (BIC) [64] was employed to allow comparison of models with different levels of complexity. Given optimised log likelihood values 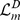 for different models 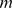 describing the same dataset, the best model was defined as that giving the *lowest* BIC value 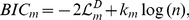
(6)where 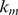 is the number of parameters included in model 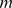, and 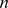 is the total number of sequences in the dataset, summed across timepoints. We note that the number of initial frequencies learnt in a calculation is derived from the properties of the data; differences in 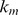 between models for a system are entirely due to the number of parameters describing selection.

Beginning with a neutral model, selection models of increasing complexity were tested, adding loci under selection. This process was continued either until adding selection to an allele at another locus did not improve the BIC value, or until the model likelihood was sufficiently close to the maximum theoretically achievable likelihood (obtained when the inferred haplotype frequencies 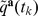 were identical to the observed frequencies 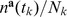) that adding an additional parameter would inevitably increase the BIC.

#### Identification of alleles potentially under selection

**Figure 4:**
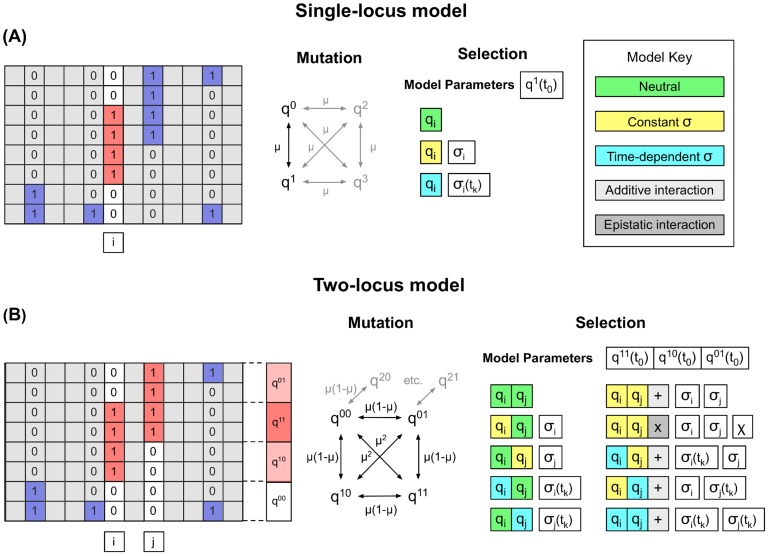
Models of selection. **(A)** Example single-locus model. A single allele, denoted 1 (red) at driver locus 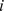 is considered to be potentially under selection; the initial consensus allele is denoted 0. Changes in the frequencies of alleles at locus 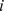 are affected over time by mutation and selection; the alleles denoted 2 and 3 refer to the remaining two nucleotides at this locus. Changes in allele frequency are compared to those obtained under models in which the allele 1 is either neutral, or under constant or time-dependent selection; appropriate parameters for selection, and for the initial state of the system, are learnt. **(B)** Example two-locus model. We suppose that alleles at loci 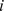 and 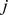 have been found to exhibit apparent non-neutral behaviour, other alleles being indistinguishable from neutrality. We divide the population into haplotypes based upon the alleles present at these loci, giving haplotype frequencies 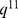, 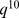, 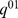, and 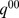. Observed frequencies are then compared to those obtained under a variety of models of selection at either one or two of these loci. In this figure, example sequences are shown for a single time-point.

The above method relies upon having a prior choice of loci in 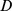, containing alleles that are potentially under selection. In the analysis conducted here, these loci were identified using a single-locus version of the above model. For each polymorphic locus, the observed allele frequencies over time were used to calculate the likelihood that the largest minority allele was under either constant or time-dependent selection, these values being compared to the likelihood of the observation under a neutral model. Likelihoods were calculated using the binomial model 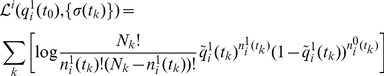
(7)where 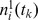 is the number of sequences observed at time 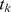 with the allele 1 at locus 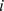, and 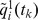 is the inferred frequency of sequences with the allele 1 at locus 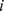 at time 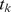. This evaluates whether or not an allele exhibits apparently non-neutral behaviour, changing in frequency either due to inherent selection, or due to linkage disequilibrium with other non-neutral alleles. All loci for which a model of non-zero selection gave a better likelihood than did a model of neutrality were included in the set 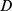. A pictorial representation of our method is given in Figure 4.

#### Describing the extent of support for a model

In the text, we describe a difference in BIC of more than 2 units as providing evidence in favour of a model, with a difference of more than 6 units providing strong evidence in favour of a model (cf. [35]). We describe a BIC difference of less than 2 units as weak evidence in favour of a model. As an additional test of the veracity of inferences from the single-locus model, we conducted bootstrapping estimates against BIC differences obtained from randomised sequence data; for all cases where we identified more than weak evidence for selection, these tests backed up our result (see Supporting Text S1).

### Validation of data

In order to test for the influence of PCR-induced recombination upon the dataset, we calculated a measure of linkage disequilibrium between loci. For each pair of polymorphic loci 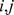 in the dataset, we calculated the value 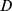, equal to the absolute linkage disequilibrium between these loci, normalised by the maximum potential linkage disequilibrium given the allele frequencies in question 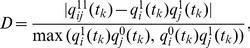
(8)where the labels 0 and 1 represent the consensus and most common minor alleles at each locus, 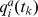 represents the frequency at time 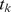 of the allele 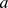 at locus 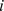, and 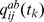 represents the frequency at time 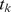 of the haplotype 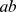 at loci 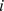 and 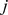. Values of 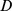 were compared for loci at different positions in the sequence, fitting a model of the form, 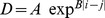 for all points for which 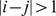, where 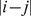 is the sequence distance between loci 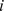 and 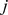. Here a greater negative value of 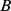 would indicate that a higher mean rate of recombination in the viral sequences occurred during the sequencing process.

### Validation of the method

A test of the ability of the method to discriminate between selected and non-selected alleles, and to correctly infer the magnitude of selection acting upon a locus, was performed by running analyses for simulated data. For simulated populations with a single allele under selection, a correlation coefficient of of more than 0.95 was found between real and inferred selection coefficients, with an equivalent correlation of 0.91 for simulated systems with two alleles under selection. Further details are given in Supporting Text S1 and Supporting Figures S6 and S7.

## Supporting Information

Figure S1 **Inferences made under the single locus method.** Model fits and corresponding log likelihoods are shown for the neutral, constant selection (*σ*), and time-dependent (*σ*(*t*)) selection models for selected loci in the data. A model of constant selection gives the optimal BIC score for Pig115 locus 188, and Pig405 locus 844. A model of time-dependent selection gives the optimal BIC score for Pig410 locus 447; the neutral model is favoured for Pig115 locus 114. Error bars give 95% posterior probability intervals for each allele frequency at each time, given the observed sequences. The optimal BIC score identified for each dataset is highlighted in bold text.
(TIF)

Figure S2 **Bootstrapping of BIC inferences.** The difference in BIC between selected and neutral models for the single allele giving the strongest evidence for selection in each animal, measured using BIC. Here a positive BIC difference shows in favour of the selected model. Values from the real sequence data are here compared to the equivalent statistic for random permutations of sequences collected from each animal. Each histogram shows the real and random statistics; a red arrow shows the position within the distribution of the real inference. In Pig104, Pig109, and Pig412, the real data gave a stronger signal of selection than all 200 random datasets. In Pig405, Pig410, and Pig115, the number of random datasets giving stronger signals of selection were one, three and eight respectively.
(PDF)

Figure S3 **Approximate locations of residues affected by nucleotide mutations in systems for which non-neutral behaviour was identified.** Residues corresponding to nucleotide polymorphisms are shown for both synonymous (orange) and non-synonymous (red) mutations. The HA1 region for one unit of the protein trimer is shown in yellow; the HA2 region, which was not included in the sequence data, is shown in blue. The two other units of the trimer are shown in grey. The residue corresponding to the nucleotide position 553 is in the Ca2 epitope site.
(PDF)

Figure S4 **No evidence found for PCR-induced recombination.** Gray dots show values of the normalised linkage disequilibrium statistic *D* for alleles at varying distances apart. The solid red line shows a sliding window average value of *D*, of width 100 bases. The dotted gray line shows the optimal fit to the data of an exponential regression line. BIC comparison of the exponential regression with a linear model favoured the latter, giving an estimate for PCR-induced recombination of zero.
(TIF)

Figure S5 **Spectrum of mutations observed in the population.** **(A)** Number of occurrences of mutations observed in the sequence data. Mutations were counted with respect to the consensus sequence, counting multiple observations of the same mutation in the same animal as a single event. **(B)** Proportion of mutations observed in the sequence data, scaled by the nucleotide content of the consensus sequence.
(PDF)

Figure S6 **Results inferred from simulated populations in which a single locus was under selection.** **(A)** True positive (red) and false positive (black) rates for identifying selection at a a selected locus, following use of the multi-locus inference model described in the main text. The blue line shows the false positive rate for identifying selection using the single-locus model; accounting for interference between alleles gives a substantially improved result. **(B)** Inferred selection coefficients obtained from the multi-locus model. Individual inferences are shown as small red circles; cases for which selection was not distinguished from neutrality are represented as having zero inferred selection. The black line is that of perfect agreement between real and inferred selection coefficients.
(TIF)

Figure S7 **Results inferred from simulated populations in which alleles at two loci evolved under additive selection.** **(A)** Combined errors in the inference of pairs of selection coefficients are shown. The error *E* in each case is calculated as the Euclidean distance between the real and inferred selection coefficients. **(B)** Inferred selection coefficients obtained from the multi-locus model for individual alleles. Inferences are shown as small red circles; cases for which selection was not distinguished from neutrality are represented as having zero inferred selection. The black line is that of perfect agreement between real and inferred selection coefficients.
(TIF)

Table S1 **Further inferences for Pig109.** The optimal model of each type is given in each case. Small BIC differences were identified between cases in which different alleles, or combination of alleles, were under selection. Model codes are *σ*: Constant selection at a single locus; 2*σ*: Additive selection at two loci. The BIC value for the optimal model is displayed in bold.
(PDF)

Text S1 **Details of optimisation of log likelihoods.** Consideration of cases of multiple alleles at single loci. Importance of mutation for inferences made for Pig109 and Pig113. Methods used in constructing Figures. Inference of selection from simulated populations. Bootstrapping via inference of selection from randomised sequence data.
(PDF)
