# Supplementary figures and images for: Identifying Selection in the Within-Host Evolution of Influenza Using Viral Sequence Data
Source: PLoS Comput Biol. 2014 Jul 31;10(7):e1003755. doi: 10.1371/journal.pcbi.1003755 (PMC4117419; doi:10.1371/journal.pcbi.1003755)

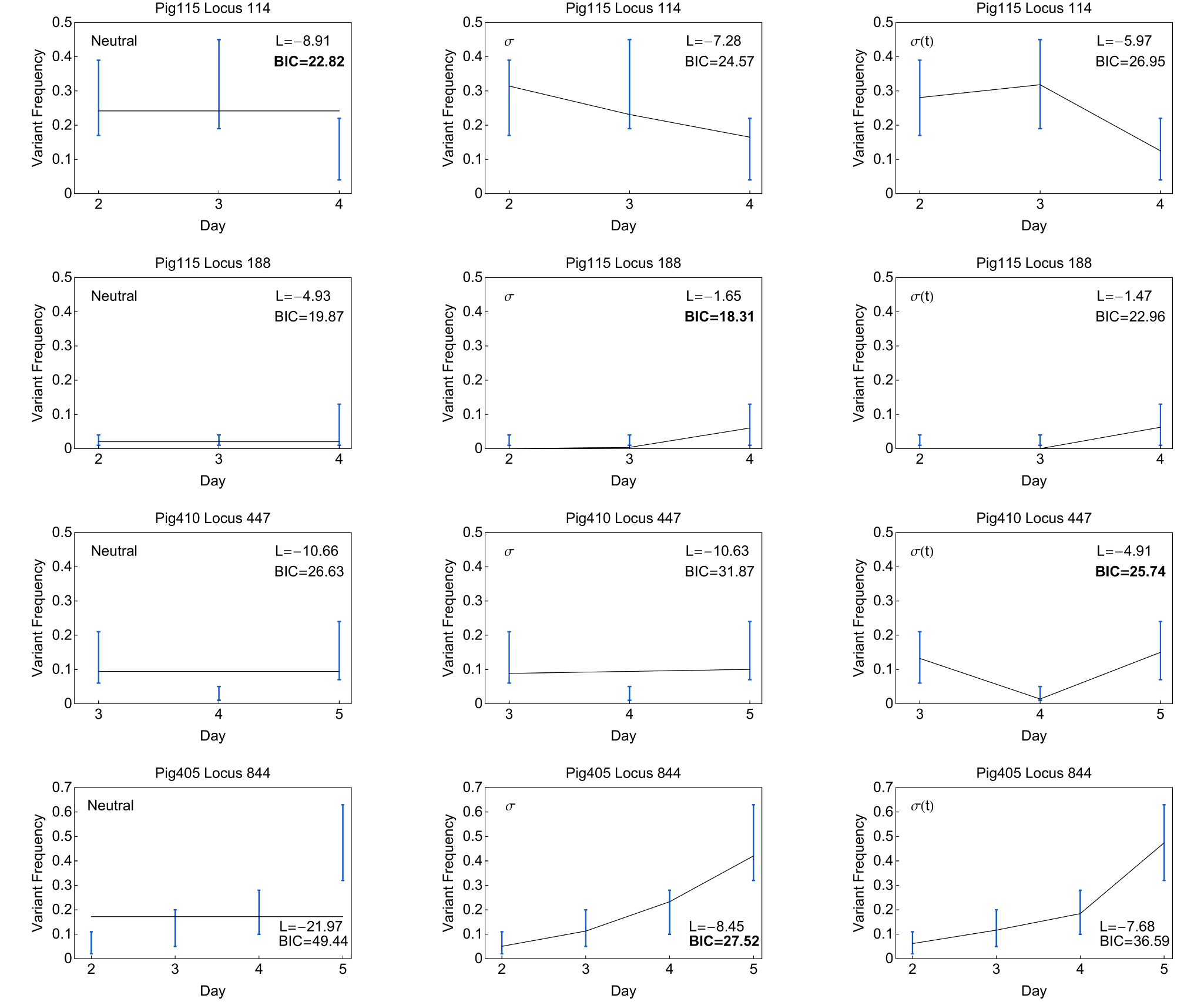

Supplement: Figure S1 — Inferences made under the single locus method. Model fits and corresponding log likelihoods are shown for the neutral, constant selection (σ), and time-dependent (σ(t)) selection models for selected loci in the data. A model of constant selection gives the optimal BIC score for Pig115 locus 188, and Pig405 locus 844. A model of time-dependent selection gives the optimal BIC score for Pig410 locus 447; the neutral model is favoured for Pig115 locus 114. Error bars give 95% posterior probability intervals for each allele frequency at each time, given the observed sequences. The optimal BIC score identified for each dataset is highlighted in bold text. (TIF) [file pcbi.1003755.s001.tif]

Pig104

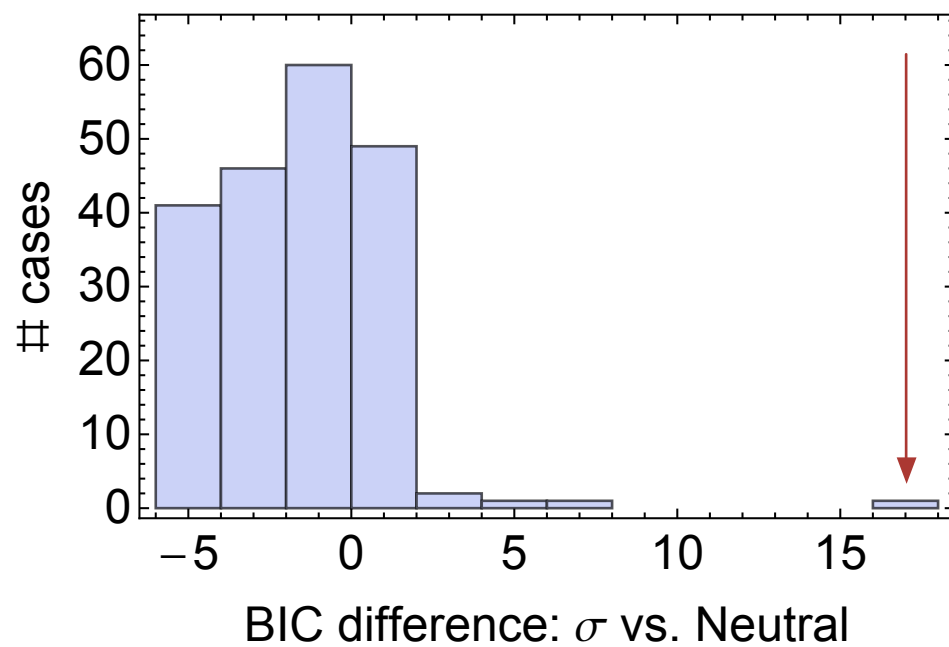

Pig109

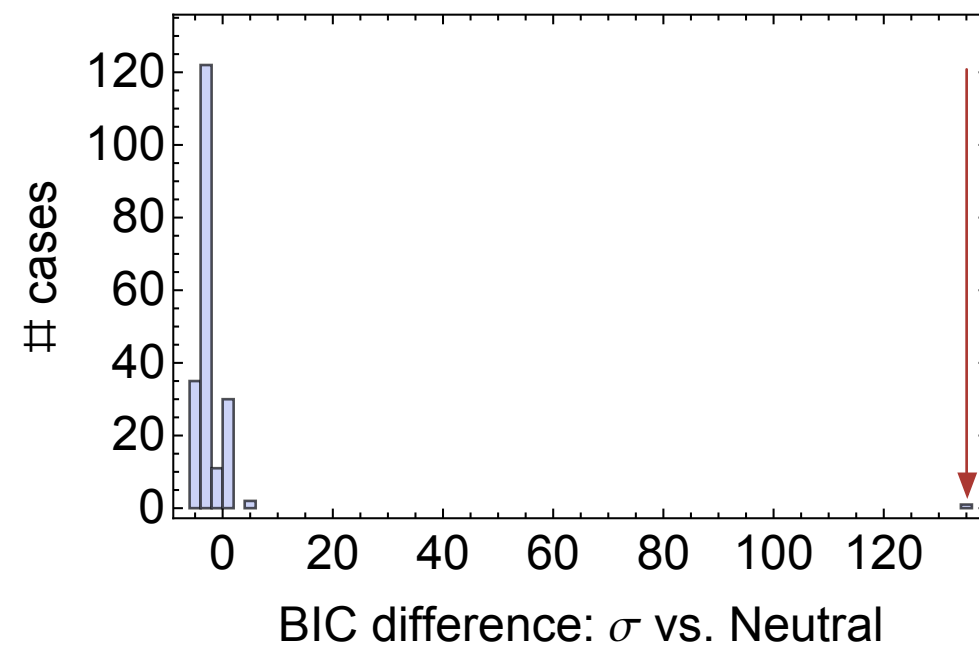

Supplement: Figure S2 — Bootstrapping of BIC inferences. The difference in BIC between selected and neutral models for the single allele giving the strongest evidence for selection in each animal, measured using BIC. Here a positive BIC difference shows in favour of the selected model. Values from the real sequence data are here compared to the equivalent statistic for random permutations of sequences collected from each animal. Each histogram shows the real and random statistics; a red arrow shows the position within the distribution of the real inference. In Pig104, Pig109, and Pig412, the real data gave a stronger signal of selection than all 200 random datasets. In Pig405, Pig410, and Pig115, the number of random datasets giving stronger signals of selection were one, three and eight respectively. (PDF) [file pcbi.1003755.s002.pdf]

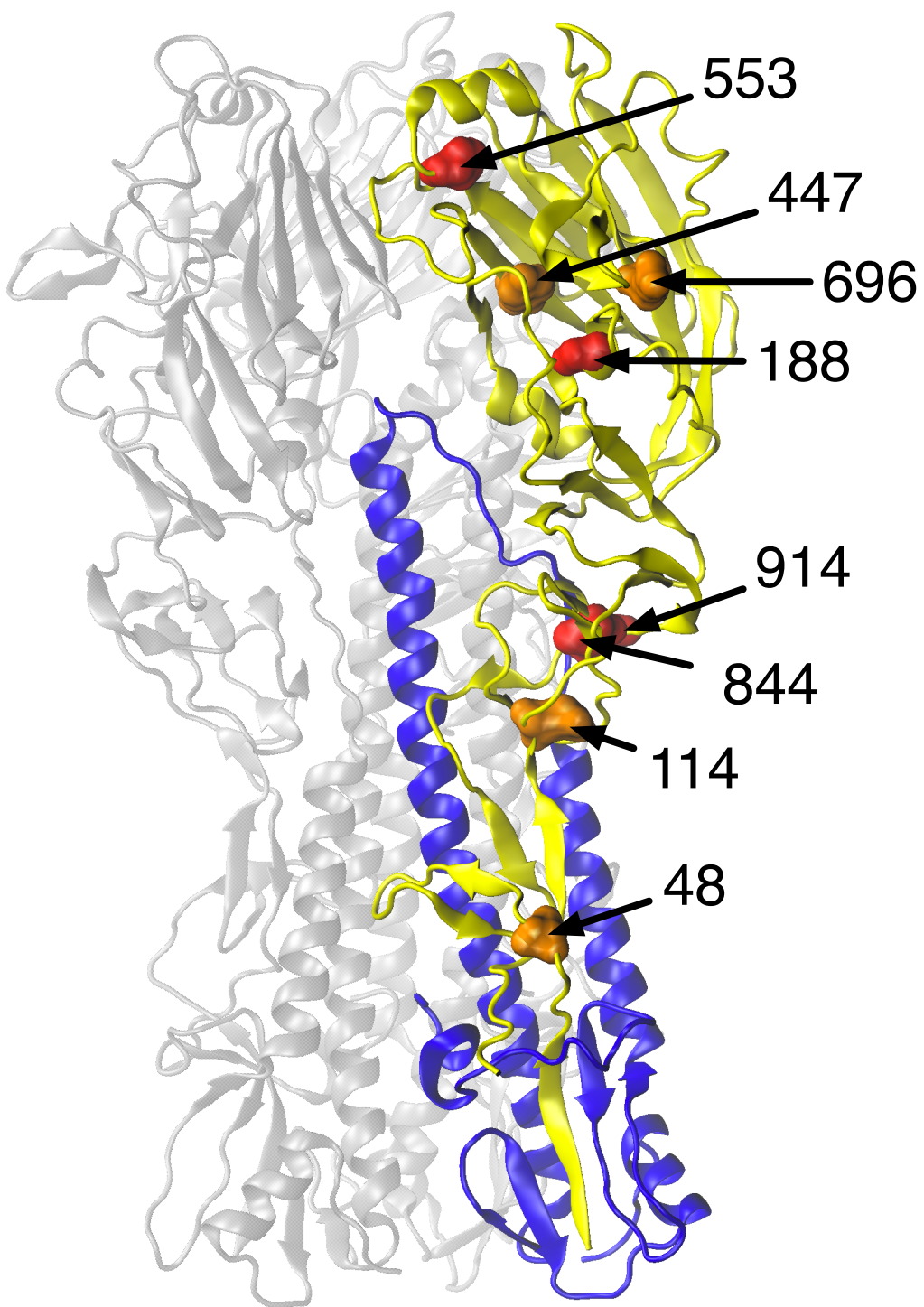

Supplement: Figure S3 — Approximate locations of residues affected by nucleotide mutations in systems for which non-neutral behaviour was identified. Residues corresponding to nucleotide polymorphisms are shown for both synonymous (orange) and non-synonymous (red) mutations. The HA1 region for one unit of the protein trimer is shown in yellow; the HA2 region, which was not included in the sequence data, is shown in blue. The two other units of the trimer are shown in grey. The residue corresponding to the nucleotide position 553 is in the Ca2 epitope site. (PDF) [file pcbi.1003755.s003.pdf]

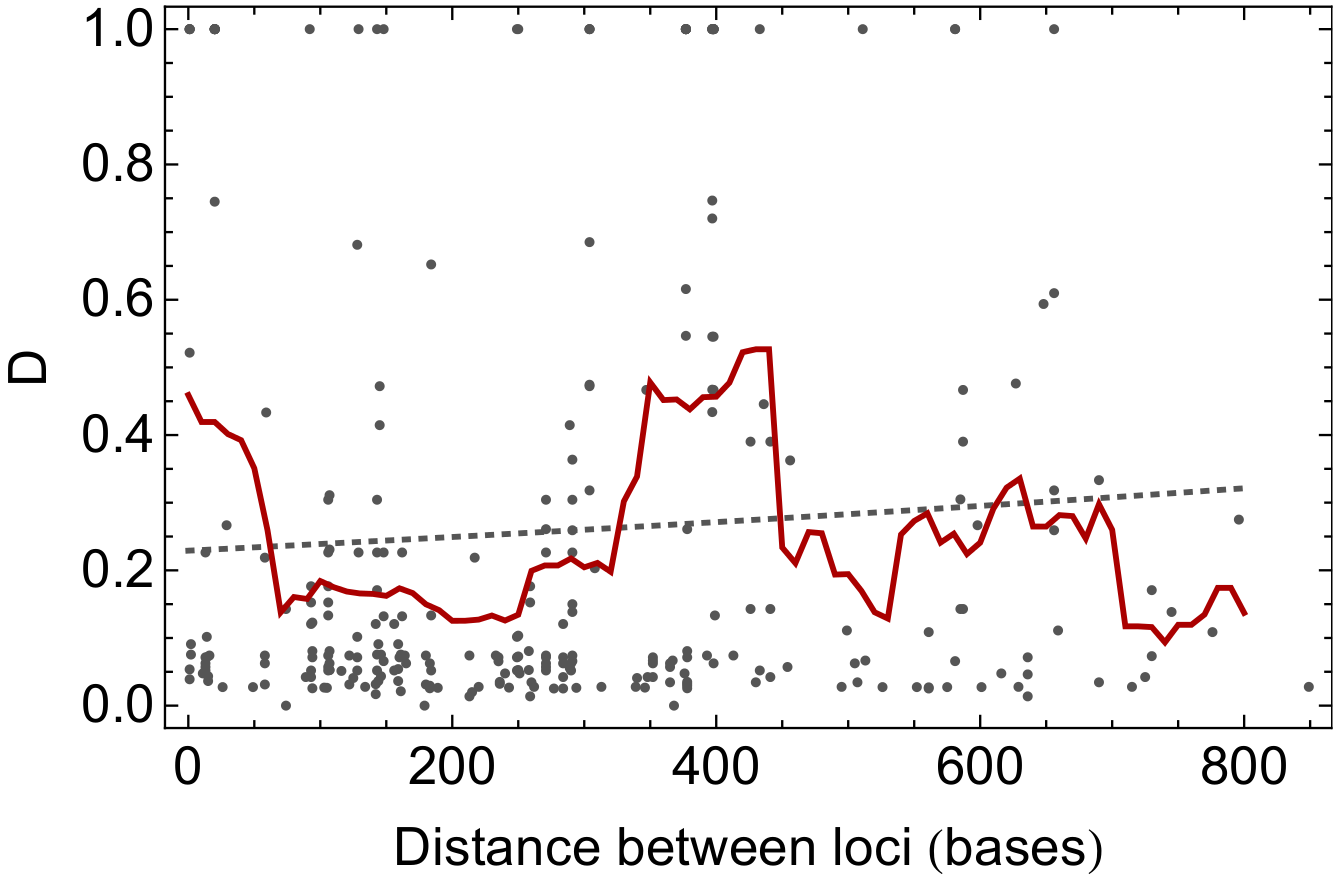

Supplement: Figure S4 — No evidence found for PCR-induced recombination. Gray dots show values of the normalised linkage disequilibrium statistic D for alleles at varying distances apart. The solid red line shows a sliding window average value of D, of width 100 bases. The dotted gray line shows the optimal fit to the data of an exponential regression line. BIC comparison of the exponential regression with a linear model favoured the latter, giving an estimate for PCR-induced recombination of zero. (TIF) [file pcbi.1003755.s004.tif]

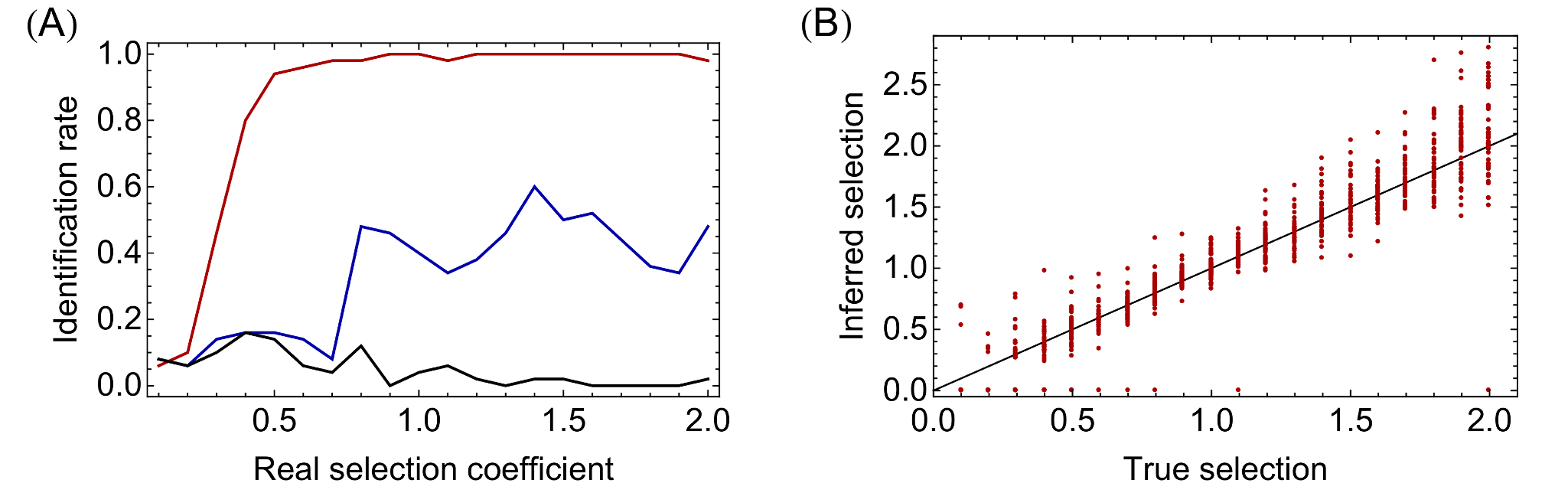

Supplement: Figure S6 — Results inferred from simulated populations in which a single locus was under selection. (A) True positive (red) and false positive (black) rates for identifying selection at a a selected locus, following use of the multi-locus inference model described in the main text. The blue line shows the false positive rate for identifying selection using the single-locus model; accounting for interference between alleles gives a substantially improved result. (B) Inferred selection coefficients obtained from the multi-locus model. Individual inferences are shown as small red circles; cases for which selection was not distinguished from neutrality are represented as having zero inferred selection. The black line is that of perfect agreement between real and inferred selection coefficients. (TIF) [file pcbi.1003755.s006.tif]

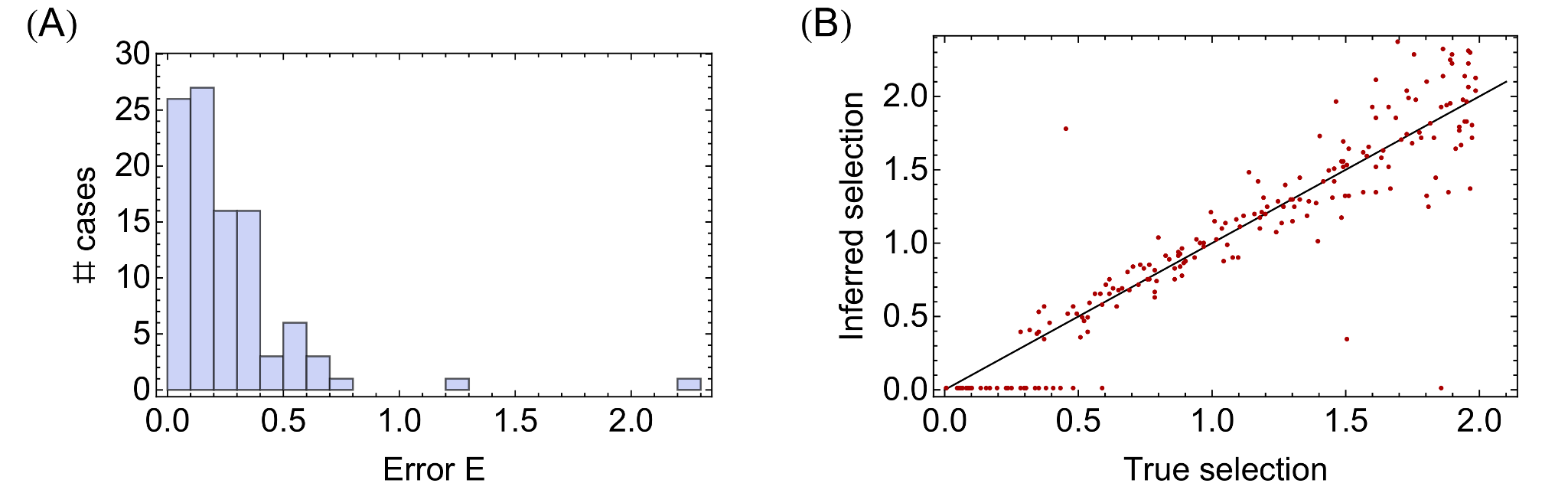

Supplement: Figure S7 — Results inferred from simulated populations in which alleles at two loci evolved under additive selection. (A) Combined errors in the inference of pairs of selection coefficients are shown. The error E in each case is calculated as the Euclidean distance between the real and inferred selection coefficients. (B) Inferred selection coefficients obtained from the multi-locus model for individual alleles. Inferences are shown as small red circles; cases for which selection was not distinguished from neutrality are represented as having zero inferred selection. The black line is that of perfect agreement between real and inferred selection coefficients. (TIF) [file pcbi.1003755.s007.tif]
