# Supplementary material for: Identifying Selection in the Within-Host Evolution of Influenza Using Viral Sequence Data
Source: PLoS Comput Biol. 2014 Jul 31;10(7):e1003755. doi: 10.1371/journal.pcbi.1003755 (PMC4117419; doi:10.1371/journal.pcbi.1003755)
Supplement: Figure S5 — Spectrum of mutations observed in the population. (A) Number of occurrences of mutations observed in the sequence data. Mutations were counted with respect to the consensus sequence, counting multiple observations of the same mutation in the same animal as a single event. (B) Proportion of mutations observed in the sequence data, scaled by the nucleotide content of the consensus sequence. (PDF) [file pcbi.1003755.s005.pdf]

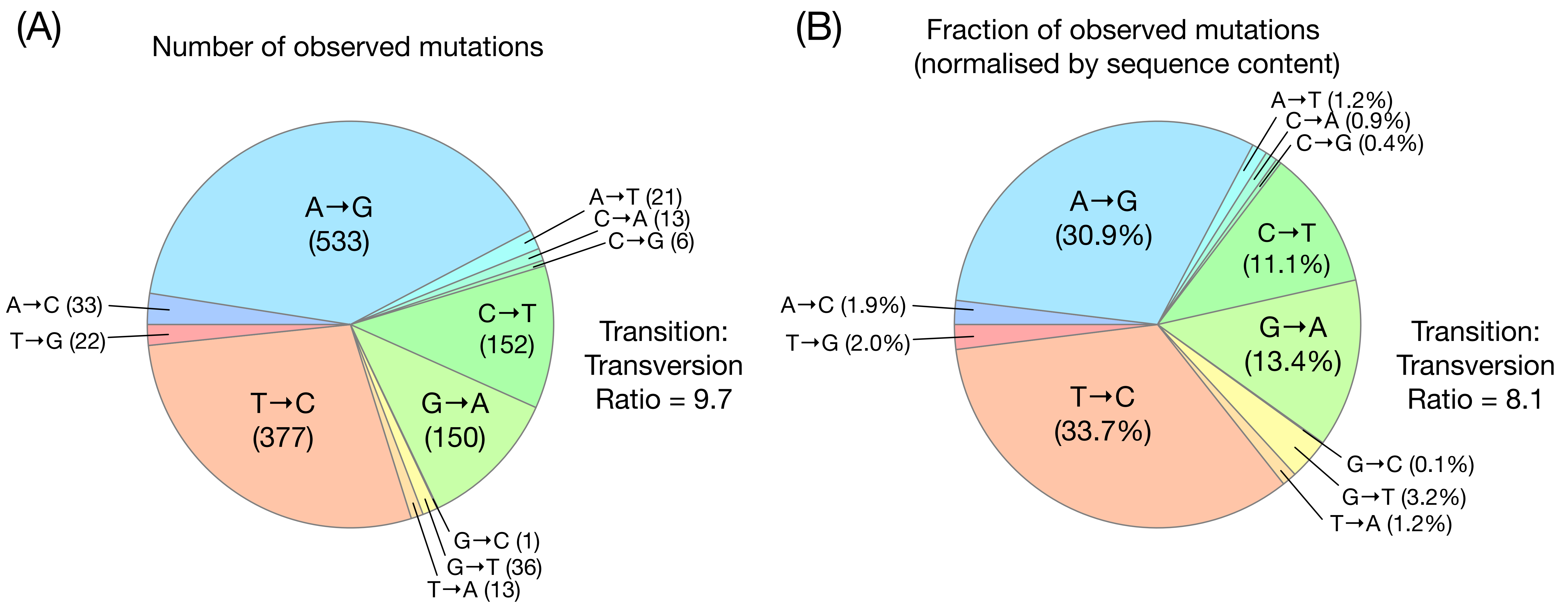
(A)
Number of observed mutations
A→G (533)
A→T (21)
C→A (13)
C→G (6)
C→T (152)
G→A (150)
G→C (1)
G→T (36)
T→A (13)
T→C (377)
T→G (22)
A→C (33)
Transition: Transversion Ratio = 9.7
(B)
Fraction of observed mutations (normalised by sequence content)
A→G (30.9%)
A→T (1.2%)
C→A (0.9%)
C→G (0.4%)
C→T (11.1%)
G→A (13.4%)
G→C (0.1%)
G→T (3.2%)
T→A (1.2%)
T→C (33.7%)
T→G (2.0%)
A→C (1.9%)
Transition: Transversion Ratio = 8.1
