# Supplementary material for: Identifying Selection in the Within-Host Evolution of Influenza Using Viral Sequence Data
Source: PLoS Comput Biol. 2014 Jul 31;10(7):e1003755. doi: 10.1371/journal.pcbi.1003755 (PMC4117419; doi:10.1371/journal.pcbi.1003755)
Supplement: Table S1 — Further inferences for Pig109. The optimal model of each type is given in each case. Small BIC differences were identified between cases in which different alleles, or combination of alleles, were under selection. Model codes are σ: Constant selection at a single locus; 2σ: Additive selection at two loci. The BIC value for the optimal model is displayed in bold. (PDF) [file pcbi.1003755.s008.pdf]

**Pig**	**Model**	**Potential driver(s)** Selection coefficients				**Log L**	**BIC**
109		**263**	**553**	**696**	**914**		
109	Neutral	0	0	0	0	-80.9	180.7
109	σ	0	0	0	3.0	-10.7	44.9
109	σ	0	0	3.0	0	-10.7	44.9
109	σ	0	3.1	0	0	-11.5	45.7
109	2σ	0	0	2.8	2.8	-3.6	**35.5**
109	2σ	0	3.0	0	3.2	-3.8	35.7
109	2σ	0	3.0	3.2	0	-3.8	35.7
