# Supplementary material for: Identifying Selection in the Within-Host Evolution of Influenza Using Viral Sequence Data
Source: PLoS Comput Biol. 2014 Jul 31;10(7):e1003755. doi: 10.1371/journal.pcbi.1003755 (PMC4117419; doi:10.1371/journal.pcbi.1003755)
Supplement: Text S1 — Details of optimisation of log likelihoods. Consideration of cases of multiple alleles at single loci. Importance of mutation for inferences made for Pig109 and Pig113. Methods used in constructing Figures. Inference of selection from simulated populations. Bootstrapping via inference of selection from randomised sequence data. (PDF) [file pcbi.1003755.s009.pdf]

Identifying selection in the intra-patient evolution of influenza using viral sequence data

Christopher J. R. Illingworth, Andrej Fischer, and Ville Mustonen

Text S1

Optimisation of log likelihoods

Maximum log likelihoods were verified by running multiple (at least 10) parallel optimisations until either the top two maximum log likelihoods were identical to three decimal places or until the difference between the top two maximum log likelihoods was substantially smaller than the BIC difference between the model under consideration and the model with the smallest BIC.

Cases of multiple alleles at a single locus

Across the whole dataset, eleven cases from six animals were found in which three separate nucleotides were observed at a single locus. However, none of the alleles in these cases were identified as potentially non-neutral using the single-locus version of our method. In other datasets, where greater evidence of non-neutrality was found for cases of multiple alleles at a locus, our method could be straightforwardly extended to account for this.

Effect of mutation: inferring selection at multiple loci in Pig109

The inference of selection made for the viral population in Pig109 sheds particular light upon the importance of mutation within our model. For example, a model in which the mutant G and A alleles at the loci 696 and 914 are under selection (with respect to the consensus alleles A and G) significantly outperforms a model in which only one of these two mutant alleles are selected for, despite the fact that these alleles are fully linked, appearing only together on a sequence, or not at all. Under a model of pure selection, this would not be the case; selection for either one allele would, via genetic hitchhiking, completely explain changes in frequency of the other.

In this case, the inference of selection upon both loci relies upon mutation. Mutation, acting upon the AG consensus, produces sequences with both the AA and GG two-locus haplotyes at these loci. Assigning selection for an A at locus 914 would increase the frequency of both the observed GA, and the unobserved AA haplotypes. In this system, the need for strong selection to increase the frequency of the GA haplotype is opposed by the need to prevent the emergence, under the model, of the AA haplotype. By contrast, in a case of additive selection for the two alleles, the double mutant GA haplotype can be assigned sufficient selection to outcompete the AA haplotype, such that while the GA haplotype increases in frequency, a much smaller increase in the AA haplotype frequency occurs as a result.

Effect of mutation: single- and multi-locus models of the population in Pig113

In the main text, we noted that, in the viral populations collected from Pig113, a very weak inference of selection was made at three loci under a single-locus model (with a BIC difference between models of 0.07 in each case), while a neutral model of evolution was preferred under a multi-locus model. This highlights a general greater conservatism inherent to the multi-locus model arising from the presence of mutation in the model.

In Pig113, sequences split between precisely two haplotypes, the consensus sequence having the alleles C, A, and G at loci 447, 824, and 844, with a minority haplotype having the alleles T, G, and A at these

loci. As such, the data in a model of three-locus haplotypes is very similar to that in the single-locus model, having two observed haplotypes, with the remainder of haplotypes unobserved. We suppose we are testing for selection acting upon the allele T at locus 447 under the single-locus, and the multi-locus models. Within the single-locus model, mutation pushes sequences from the C and T alleles to the A and G alleles, increasing their frequency, while selection acts purely on the T allele with respect to the A, C, and G alleles. Within the multi-locus model, however, there is mutation out of the initial CAG and TGA haplotypes to many other haplotypes, including those, such as TGG, and TAA, which have the T allele at locus 447. Under selection each of these haplotypes will also grow in frequency over time, yet these haplotypes are not observed in the data. This non-observation of selected haplotypes restricts the magnitude of selection that can be assigned to the T allele, leading to a lower inferred selection coefficient, a smaller difference between the likelihoods of the neutral and selected model, and a lower probability of inferring selection at this locus. Thus, while the focus of discussion about our model is on the importance of selection, in cases such as this and the above, mutation may have an impact, and it cannot be excluded from the model.

Construction of figures

Error bars in Figure 3

The error bars shown in Figure 3 of the main text give an approximate representation of the uncertainty inherent to each observation, the true values being multidimensional in form. Error bars were calculated from marginal posterior probabilities for each allele frequency, given the observation. The prior distribution for each of these was set as the beta distribution $\mathrm{Beta}(\epsilon, \epsilon)$, where $\epsilon = 0.01$, reflecting a state in which an allele is likely to be close to fixation. Where the alleles 1 and 0 were observed n_1 and n_0 times respectively, this gives the posterior distribution $\mathrm{Beta}(n_1 + \epsilon, n_0 + \epsilon)$. Calculating this distribution on a 1% frequency interval grid, the error bar was defined as the minimum interval around the mode of this distribution containing at least 95% of the distribution.

Visualisation of mutations in the HA structure

The protein structure in the PDB database with the closest sequence identity to the sequence of HA gene of the influenza virus used in the study was identified via a BLAST search as 2WRH, with 86% sequence identity [1]. The locations of apparently non-neutral mutations were mapped onto this structure, and are displayed in Supporting Figure S3 using VMD, in conjunction with the SURF and STRIDE packages [2–4].

Calculation of the mutational spectrum

Within the data for each animal, occurrences of non-consensus alleles were counted. Where the same non-consensus allele was observed more than once in a single animal, the assumption was made that the allele had arisen through a single mutation event, being observed more than once in the population as a result of selection and sampling effects. This approximation attempts to account for bias in the spectrum caused by positive selection acting upon a few specific alleles. Details are shown in Supporting Figure S5.

Inference of selection from simulated populations

The inference method was applied to data from simulated populations in order to estimate the accuracy with which selection was inferred, and to evaluate the extent to which the multi-locus model outperformed a model of evolution at individual loci.

Selection acting upon a single allele

In the observed sequences, a total of 3005 non-consensus alleles were observed from a total of just over 2.4 million nucleotide reads, giving a mean frequency of polymorphism close to 0.125%. While potentially non-neutral alleles in the study had higher mean frequencies than this, we conducted simulations under the expectation that a population with initially low sequence diversity would evolve, subject to mutation and selection. As such, a population was defined consisting of 10^6 sequences each with ten two-allele loci, alleles being denoted 0 and 1. An initial population was created by assigning the allele 1 at each locus to $2r\%$ of randomly chosen sequences, where r was drawn independently for each locus from the uniform distribution on the interval $[0, 1]$. The alternative allele at the first locus was given a fixed selective advantage s, and the population was propagated using a Wright-Fisher model for 16 generations. Every four generations, beginning at the fourth generation, a sample from the population of size 100 sequences was taken, giving data from four time points; for the purposes of the inference, a generation was assumed to last six hours. Fifty simulations were generated for each of twenty different values of s, giving one thousand simulations in total.

For each simulation, the method described in the main text was applied to generate an inference of selection. Firstly, a single-locus model was applied to each locus at which polymorphism was observed. Models of allele dynamics in which the minority allele at the locus evolved neutrally, or was under constant or time-dependent selection were evaluated using a binomial likelihood function (Equation 7 of the main text), using the Bayesian Information Criterion (BIC) to compare models, and hence to identify alleles that exhibited apparently non-neutral behaviour. Next, in cases for which more than one allele was apparently non-neutral, a multi-locus model was applied so as to separate alleles which changed in frequency due to inherent selection from those which changed in frequency due to interference from other alleles. Inferred selection coefficients, and true and false positive rates of identifying loci with alleles under selection, were than calculated.

In general, our method performed well in identifying alleles under selection. Above a selection coefficient of 0.5 $(12h)^{-1}$, alleles under selection were identified as being such in more than 90% of simulations. Further, at these selection coefficients, the mean inferred value of selection was very close to the real value, sampling noise providing one explanation for deviation from the mean in particular inferences. The mean error in the selection coefficient inferred for an allele under selection was 0.14 $(12h)^{-1}$, treating cases for which selection was not identified as having an inferred selection coefficient of zero.

Our simulations also highlighted the need for an account to be taken of interference effects between alleles. Across the 1000 simulations, a total of 329 alleles were falsely identified to be significantly non-neutral using a single-locus inference approach. When a multi-locus model was applied, this figure fell substantially, to 47 false positive inferences. Results are shown in Supporting Figure S6.

Selection acting upon alleles at two loci

Further simulations were conducted for a system identical to that above in which alleles at two loci were under additive selection, with selection coefficients σ_1 and σ_2 chosen to be uniform random variables between 0 and 2 $(12h)^{-1}$. Again, potentially non-neutral alleles were identified using a single-locus model, before applying a multi-locus model to infer selection coefficients. Given inferred selection coefficients $\tilde{\sigma}_1$ and $\tilde{\sigma}_2$, the error in the inference E was calculated as the Euclidean distance between the real and inferred coefficients:

$$E = \sqrt{(\sigma_1 - \tilde{\sigma}_1)^2 + (\sigma_2 - \tilde{\sigma}_2)^2} \quad (1)$$

A total of 100 populations were simulated with additive two-locus selection. Real and inferred se-

lection coefficients, and error values E are shown in Supporting Figure S7. Across the simulations, the mean value of E was 0.26 $(12h)^{-1}$, about one quarter of the mean strength of selection acting upon a selected allele. The worst error values arose when selection was incorrectly assigned to the wrong locus. The true positive rate of identifying alleles under selection was 85%.

Errors in our inferences can generally be attributed to sampling noise. At the lowest selection coefficients, selection does not change the allele frequencies enough to be observed at the given depth of sampling, such that sampling noise becomes the main determinant of any inferred selection coefficient. At higher selection coefficients, sampling also becomes an issue; by the time of the first sampling point, the selected allele frequency has already reached a substantial value, while the allele is usually fixed by the time of the second sampling point; the time-resolution at which samples are collected is too long to obtain accurate results. In the inferences of selection at a single locus, we thus observe a sweet-spot at a coefficient of around 1 per 12 hours, where the variance in inferred selection is at its smallest.

We note one limitation to our simulation approach. Whereas in the simulated data, the population structure and the mechanisms by which it evolves mirror those used in the inference, the actual structure of a viral population evolving within an animal may be more complex. Our inferences from simulated populations should therefore be considered as illustrative of the performance of the method, rather than strict benchmark calculations.

Inference of selection from randomised sequence data

In a further test, we applied our method to random permutations of sequence data. Sequences were chosen from the pigs for which selection was detected in the original analysis. For each pig, sequence data were shuffled in time, preserving the number of sequences assigned to each time point, but assigning individual sequences to random time-points. Two hundred random sets of sequence data were generated in each case. Next, for each polymorphic locus in each randomly-generated set for which at least two mutant alleles were observed at a given time-point, a single-locus inference of selection was conducted, calculating BIC scores for a neutral model, and for models of constant and time-dependent selection. The largest BIC score difference for each random set of data was then compared to the corresponding BIC difference inferred for the real sequence data. This bootstrapping provides an additional test for the veracity of the inference of selection, additional to the use of BIC itself.

In three out of the six animals, Pig104, Pig109, and Pig412, the maximum BIC difference collected from the random data was substantially lower than the corresponding value obtained from the real sequence data (Supporting Figure S2). In the remaining animals, the BIC difference was lower than the great majority of random sets, with eight, one, and three random sets respectively obtaining higher BIC differences than the result obtained for the real data for Pig115, Pig405, and Pig410. These results support the idea that the selection we identify in the viral populations is caused by real frequency changes, rather than stochastic fluctuations in the data.

References

1. Liu J, Stevens DJ, Haire LF, Walker PA, Coombs PJ, et al. (2009) Structures of receptor complexes formed by hemagglutinins from the asian influenza pandemic of 1957. Proc Natl Acad Sci USA 106: 17175–80.

2. Humphrey W, Dalke A, Schulten K (1996) VMD - Visual Molecular Dynamics. J Molec Graphics 14: 33–38.

3. Frishman D, Argos P (1995) Knowledge-based secondary structure assignment. Proteins: structure, function and genetics 23: 566–579.

4. Varshney A, Brooks FP, Wright WV (1994) Linearly scalable computation of smooth molecular surfaces. IEEE Comp Graphics and Applications 14: 19–25.
